# A quantum annealing architecture with all-to-all connectivity from local interactions

**DOI:** 10.1126/sciadv.1500838

**Published:** 2015-10-23

**Authors:** Wolfgang Lechner, Philipp Hauke, Peter Zoller

**Affiliations:** 1 Institute for Quantum Optics and Quantum Information, Austrian Academy of Sciences, 6020 Innsbruck, Austria.; 2 Institute for Theoretical Physics, University of Innsbruck, 6020 Innsbruck, Austria.

**Keywords:** Quantum mechanics, Adiabatic Quantum Annealing, Quantum Computing

## Abstract

Quantum annealers are physical devices that aim at solving NP-complete optimization problems by exploiting quantum mechanics. The basic principle of quantum annealing is to encode the optimization problem in Ising interactions between quantum bits (qubits). A fundamental challenge in building a fully programmable quantum annealer is the competing requirements of full controllable all-to-all connectivity and the quasi-locality of the interactions between physical qubits. We present a scalable architecture with full connectivity, which can be implemented with local interactions only. The input of the optimization problem is encoded in local fields acting on an extended set of physical qubits. The output is—in the spirit of topological quantum memories—redundantly encoded in the physical qubits, resulting in an intrinsic fault tolerance. Our model can be understood as a lattice gauge theory, where long-range interactions are mediated by gauge constraints. The architecture can be realized on various platforms with local controllability, including superconducting qubits, NV-centers, quantum dots, and atomic systems.

## Teaser

An architecture for adiabatic quantum computing devices, which is programmed by local fields and features all-to-all connectivity.

The step from mechanical calculators that perform hardwired operations to fully programmable computers initiated the information age several decades ago. With the remarkable progress in preparation and control of quantum bits (qubits), we are now approaching the age of quantum information. The step to a programmable, scalable, and universal quantum device is the current challenge across platforms and disciplines. A promising route of using quantum computing technologies in practical applications is quantum annealing (*1*–*3*). Quantum annealing is a finite-temperature protocol based on adiabatic quantum optimization (*1*) with the aim to efficiently solve optimization problems. The existence of a quantum speedup in an actual device with finite qubit lifetimes, temperature, and other sources of errors compared to classical algorithms is currently the subject of lively debate (*4*–*8*). With the prospect of overcoming these technical challenges in next-generation devices, quantum annealing is currently gaining attention in academia and industry (*2*, *3*, *9*–*11*).

**Fig. 1:**
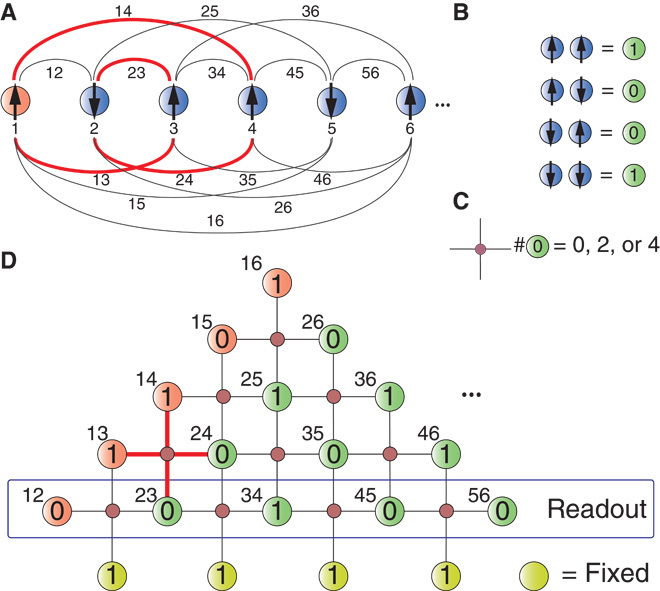
Illustration of the fully connected architecture. (**A**) The aim is to encode a system of *N* logical spins with programmable infinite-range interactions (solid lines). (**B**) New physical qubit variables are introduced for each of the *N*(*N* − 1)/2 interactions, which take the value 1 if two connected logical spins point in the same direction and 0 otherwise. (**C**) The new physical qubits are noninteracting except for local constraints on plaquettes of four spins. (**D**) The constraints correspond to closed paths connecting logical spins [for example, the red cross in (D) corresponds to the red lines in (A)]. The number of 0’s in a plaquette can be either 0, 2, or 4. The particular arrangement of new spins shown in (D) allows for a two-dimensional representation of the infinite-range model with local constraints only. An additional row of physical qubits fixed to 1 (yellow) completes the implementation. The solution of the optimization problem can be read out in specific combinations of the physical qubits, for example, as marked in (D).

The paradigm of quantum annealing is to encode an optimization problem in the interactions between classical variables that can take the values ±1. Thus, the problem is cast into the form (depicted in Fig. 1A) of an all-to-all Ising spin glass model (*12*)

$$H_{f}=\sum_{i=1}^{N}\sum_{j<i}J_{ij}\sigma_{z}^{\left(i\right)}\sigma_{z}^{\left(j\right)}+\sum_{i=1}^{N}b_{i}\sigma_{z}^{\left(i\right)}$$ (1)

where σ_*z*_^(*i*)^ is the *z*-Pauli matrix associated with the *i*th spin. The interaction matrix *J*_*ij*_ and the additional local magnetic fields *b*_*i*_ fully parameterize the optimization problem. The task of finding the optimal solution amounts to finding the ground state of *H*_*f*_. Adiabatic quantum annealing aims at achieving this by turning the classical spin variables into qubits and adiabatically transferring the system from a trivial initial state, for example, the ground state of *H*_0_ = ∑_*i*_*h*_*i*_σ_*x*_^(*i*)^, to the ground state of *H*_*f*_. The protocol is executed by the time-dependent Hamiltonian

$$H(t)=A(t)H_{0}+B(t)H_{f}$$ (2)

with *A* = 1 and *B* = 0 initially and *A* = 0 and *B* = 1 at the end of the sweep. If the sweep is sufficiently slow, the quantum annealer reaches the ground state of *H*_*f*_ with the aid of quantum tunneling, and one has thus found the desired result of the optimization.

To achieve a universal quantum annealer in the spin glass picture, each element of *J*_*ij*_ has to be controllable. However, the interactions of physical qubits are fundamentally quasi-local by nature, which severely restricts the control over the elements in the interaction matrix *J*_*ij*_. For example, in quantum annealers such as the D-Wave machine (*2*, *3*), particular pair interactions are hardwired, whereas in other potential realizations, such as ultracold gases in optical lattices, interactions would be determined by the geometry (*13*, *14*).

Here, we present an approach that overcomes this challenge by detaching from the spin glass paradigm. Our architecture relies on a conceptual division between the logical qubits σ, defining the problem given in Eq. 1, and the physical qubits ${\tilde{\sigma }}_{z}$ available in the laboratory. To differentiate the notation, we call the eigenstates of $\tilde{\sigma }$ for the physical qubits 1〉, 0〉 with eigenvalues +1, −1. The physical qubits represent the relative configuration of two logical qubits along a given bond *J*_*ij*_, with a parallel (antiparallel) alignment being mapped to 1 (0) (see Fig. 1B). The optimization parameters *J*_*ij*_ then become, in the laboratory, local magnetic fields, allowing the user to fully program the device with local control. As discussed in detail below, the logical qubits are redundantly encoded in the topology of the new architecture, enabling an intrinsic fault tolerance of the device. Moreover, the architecture can be generalized to encoding single-particle and general *n*-body interaction terms. To accommodate all interaction matrix elements, the system size in our architecture is enlarged from *N* logical qubits to *K* = *N*(*N* − 1)∕2 physical qubits. This increased number of degrees of freedom is compensated by *K* − *N* + 1 constraints *C*_*l*_, which one can realize with local interactions in a simple square-lattice geometry.

The optimization problem is encoded in the Hamiltonian

$$H_{p}=\sum_{k=1}^{K}J_{k}{\tilde{\sigma }}_{z}^{\left(k\right)}+\sum_{l=1}^{K-N+1}C_{l}$$ (3)

The vector *J*_*k*_ runs over all *K* = *N*(*N* − 1)∕2 elements of the interaction matrix *J*_*ij*_ from Eq. 1, thus translating the optimization parameters into easily controllable local fields that act on physical qubits. Below, we show that with the adequate choice of the constraints and their geometrical arrangement, all *n*-body interactions (local magnetic field, pair interactions, three-body interactions, etc.) can be encoded in Eq. 3.

The constraints *C*_*l*_ are constructed from conditions on closed loops of logical qubits with the necessary requirements (i) that the constraints cover all physical qubits and (ii) that the number of constraints is at least *K* − *N*. As an illustrative example for two-body terms, consider the closed loop of four bonds σ_*z*_^(1)^σ_*z*_^(3)^→σ_*z*_^(2)^σ_*z*_^(3)^→σ_*z*_^(2)^σ_*z*_^(4)^→σ_*z*_^(1)^σ_*z*_^(4)^ (red lines in Fig. 1A). Consistency of the relative alignment of σ_*z*_^1,2,3,4^ demands either none, two, or all four of the pairs of logical spins to be antiparallel. That is, the number of 1’s in the four physical qubits ${\tilde{\sigma }}_{z}^{13},{\tilde{\sigma }}_{z}^{23},{\tilde{\sigma }}_{z}^{24},{\tilde{\sigma }}_{z}^{14}$ has to be even (red cross in Fig. 1D). The same considerations apply for any closed loop in the logical qubits. For example, along a closed triangle, the number of physical qubits equal to 0 can be 0 or 2. Similar constraints are also relevant in the context of lattice gauge theories (*15*). From all the possible closed loops, we select those that (iii) can be implemented in real space on a simple geometry with local interactions only.

The solution that satisfies all the above conditions (i) to (iii) is illustrated in Fig. 1D. For this, the constraints are constructed as follows: Consider the index distance between logical qubits *s* = |*i* − *j*|. The chosen loops consist of four connections: one of index distance *s*, two connections with distance *s* + 1, and one with distance *s* + 2. As an illustration, a building block loop with *s* = 1 is shown in Fig. 1 (A and D) marked in red. The total of all *s* = 1 loops gives *N* − 3 constraints. The next building block is a loop with *s* = 2, which can be geometrically added as an additional row in a triangle, as shown in Fig. 1D. Continuing this procedure up to *s* = *N* − 2 results in a construction that satisfies all conditions (i) to (iii).

In a physical device, the local constraints can be enforced in various possible ways. Two typical forms to write such constraints are

$$\begin{array}{ccc} C_{l} & = & +C{\left(\sum_{m=n,e,s,w}{\tilde{\sigma }}_{z}^{\left(l,m\right)}+S_{z}^{l}\right)}^{2}\;\text{or}\\ C_{l} & = & -C{\tilde{\sigma }}_{z}^{\left(l,n\right)}{\tilde{\sigma }}_{z}^{\left(l,e\right)}{\tilde{\sigma }}_{z}^{\left(l,s\right)}{\tilde{\sigma }}_{z}^{\left(l,w\right)} \end{array}$$ (4)

Here, the first sum represents an “ancilla-based” implementation. The sum runs over the four members of each plaquette (north, east, south, and west) and *S*_*z*_ is an ancilla qutrit with three possible values: −4, 0, or 4. Implementations with ancilla qubits can also be implemented with qubits only. The second form is an implementation that requires a four-body interaction on the plaquettes. The preferable implementation of the constraints depends on the details of the physical qubits (for example, superconducting qubits, cold atoms, molecules or ions, and cavities).

As a final step, the boundaries of the lattice of physical qubits have to be taken care of. In Fig. 1D, the bottom row (labeled with “Readout”) consists of triangles instead of squares. These can be treated in two ways: (i) introduce a separate constraint enforcing the condition that the number of 0’s in each of these triangles is odd and (ii) introduce *N* − 2 additional physical qubits that are fixed to 1 as shown in Fig. 1D. The latter realization has the advantage that all constraints in the resulting square lattice are treated on the same footing. The entire scheme is scalable in a natural way: adding one logical qubit is equivalent to adding a “line” of *N* physical qubits to the new system.

The protocol to find the ground state of the new Hamiltonian is the same as in the original Ising spin glass quantum annealing described in Eq. 2. As an initial state for the optimization protocol, we choose the ground state of a simple Hamiltonian that can be adiabatically transformed into Eq. 3. Note that other choices of initial states may improve the protocol. The simplest form for illustration could be

$$H_{p0}=\sum_{k=1}^{K}h_{k}{\tilde{\sigma }}_{x}^{\left(k\right)}$$ (5)

where the sum runs over all *K* new degrees of freedom. In the realization of *C*_*l*_ based on ancillas, these have to be also included in *H*_*p*0_. The adiabatic sweep is described by the time-dependent Hamiltonian

$$H_{\text{prog}}(t)=A(t)H_{p0}+B(t)H_{p}$$ (6)

Note that in this architecture, the local field term and constraint term in *H*_*p*_ can be independently switched. A particularly useful implementation could be to leave the interactions constant during the sweep. This concludes the architecture of a fully connected spin model that is programmable with local fields.

**Fig. 2:**
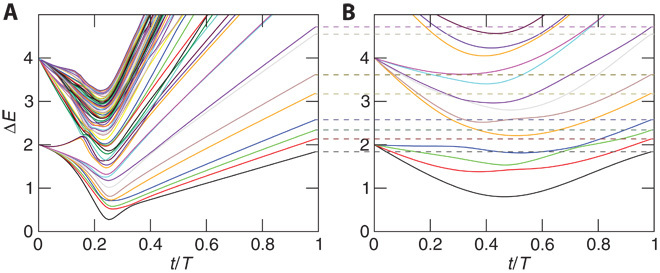
Time-dependent spectrum. (**A** and **B**) Energy spectrum of a typical adiabatic sweep with *N* = 4 logical qubits and an additional random field in the programmable implementation (A) and in a fictitious implementation of the logical qubits (B). Here, *t* is the time and *T* is the total time of the sweep. Instantaneous eigenenergies *E*_*i*_ are measured with respect to the ground state, Δ*E* = *E*_*i*_ − *E*_0_. The constraint strength is *C/J* = 2, and the elements of the *J*_*ij*_ matrix are random numbers uniformly taken from the interval [−*J*,*J*]. Although the adiabatic transformation follows different quantum paths, at the end of the sweep an exact correspondence between the lowest levels of the programmable architecture and the original model of classical spins is achieved (dashed lines).

The new time-dependent Hamiltonian is embedded in a larger Hilbert space and has a different spectrum, and the sweep is associated with a different phase transition compared to the adiabatic optimization in Eq. 2. The difference between the two sweeps is illustrated in Fig. 2. This difference in the quantum path during the sweep may also offer interesting new opportunities to approach challenges in quantum annealing, including the question of the scaling of the minimal gap or the role of temperature and errors from qubit imperfections.

The lowest states in the final Hamiltonian are identical in both representations of the optimization problem. During the sweep, the minimal gap in the programmable model is smaller than that in the fictitious direct implementation of the logical model. However, it may scale similar to or better than a realistic implementation of such a fully connected graph, which is realized by embedding (*16*, *17*) in a large number of highly connected subgraphs. Note that the constraints are fundamentally different from the ferromagnetic chains in embedding strategies (*16*, *17*). The role of the two-dimensional nature of the plaquette constraints during the sweep is an open question.

For details on the static errors from finite *C*, see fig. S1. A comparison of the success probability for a small system between fictitious all-to-all spin glass and programmable architecture is depicted in fig. S2. Some special cases with a straightforward geometrical interpretation in the programmable architecture are depicted in fig. S3.

## MATERIALS AND METHODS

**Fig. 3:**
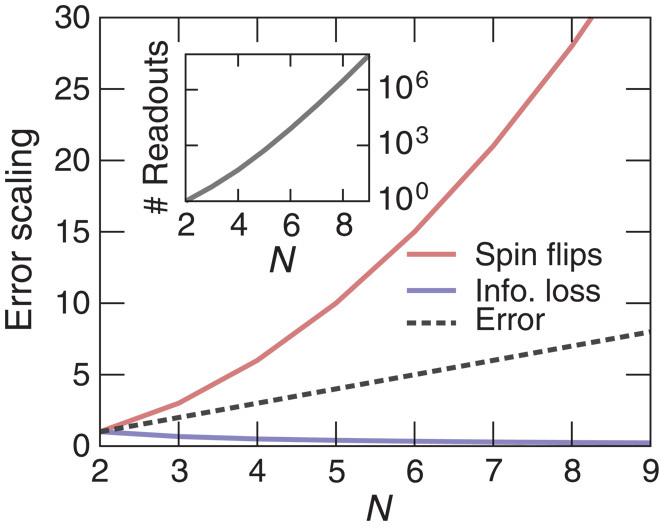
Error tolerance. The probability for a spin flip due to decoherence increases with the number of physical qubits (quadratically with *N*) (red). The information loss per physical qubit decreases with *N* (blue) and compensates the increased spin flips such that the total error scales linearly (dashed). The number of possible readout sequences (inset) exponentially increases with *N*.

The parity-based architecture includes an intrinsic fault tolerance, with some similarities to the error robustness of topological quantum memories (*18*). As a result, the error due to spin flips scales linearly with the number of logical qubits and not with its square. The reason is a redundant encoding of the information about the logical qubits in the physical qubits. The solution of the optimization is fully determined by reading out an adequate choice of *N* − 1 among the *N*(*N* − 1)∕2 physical qubits. An example is marked in Fig. 1D: the relative configurations of the pairs 12, 23, 34, 45, and 56 fully determine the logical spins up to a global inversion. Other combinations of pairs hold the same information. As shown in the inset of Fig. 3, the total number of possible readouts (the “determining combinations”) exponentially increases with *N*.

To quantify the fault tolerance, the question we want to address is: what is the probability of retrieving the correct result from reading out all possible determining combinations even if a spin flipped as a result of decoherence? More formally, this is the probability *P*_*d*_ that an independent spin flip occurs during the adiabatic passage multiplied by the probability *P*_*m*_ that a measurement indicates an erroneous solution due to this error. The latter is related to the information loss from a single spin flip.

In a fictitious implementation of the fully connected spin glass model, the probability that a spin flips because of decoherence is estimated as *P*_*d*_ = *N*Γ*T*, where *N* is the number of spins, Γ is the decoherence rate, and *T* is the total time of the adiabatic passage. The probability of measuring the wrong result if a spin has flipped is *P*_*m*_ = 1. Therefore, decoherence linearly suppresses the success probability with *N*.

In our architecture, the number of physical qubits is *N*(*N* − 1)∕2. Assuming the same qubit quality, the probability *P*_*d*_ = *N*(*N* − 1)∕2Γ*T* now scales with *N*^2^ (see Fig. 3, red). However, the information content of a single physical spin is given by the ratio of determining readouts that contain the given spin, *N*_*f*_, to the total number of possible determining readouts *N*_meas_, *P*_*m*_ = *N*_*f*_∕*N*_meas_ (see Fig. 3, blue). Remarkably, the product

$$P_{d}P_{m}=\frac{N(N-1)}{2}\Gamma TP_{m}=(N-1)\Gamma T$$ (7)

is identical to the error scaling in the original spin model. The redundancy in the measurement exactly compensates the increased error rate from single spin flips. That is, because *P*_*m*_ = 2∕*N*, a majority-vote readout will give the correct answer as long as less than *N*∕4 physical qubits are compromised.

The proposed scheme can be generalized from two-body interactions (Eq. 1) to the inclusion of single-qubit and higher-order interaction terms. Single-qubit terms, equivalent to an additional magnetic field acting on the logical qubits, can be simply realized by adding an auxiliary logical spin, say *i* = 1, that is fixed to σ_*z*_^(1)^ = +1. Its interaction with the remaining particles realizes the desired field terms, ∑_*i*_*b*_*i*_σ_*z*_^(*i*)^ = ∑_*i*_*J*_*i,*1_σ_*z*_^(*i*)^σ_*z*_^(1)^, with *J*_*i,*1_ = *b*_*i*_ (red qubits in Fig. 1D). In the programmable system, this additional spin can be included by the addition of another row of physical qubits.

Higher-order interactions can be implemented with the same local constraints in higher dimensions. Consider a three-body Hamiltonian

$$H_{p}=\sum_{i}\sum_{i<j}\sum_{i<j<k}J_{ijk}\sigma_{z}^{\left(i\right)}\sigma_{z}^{\left(j\right)}\sigma_{z}^{\left(k\right)}$$ (8)

**Fig. 4:**
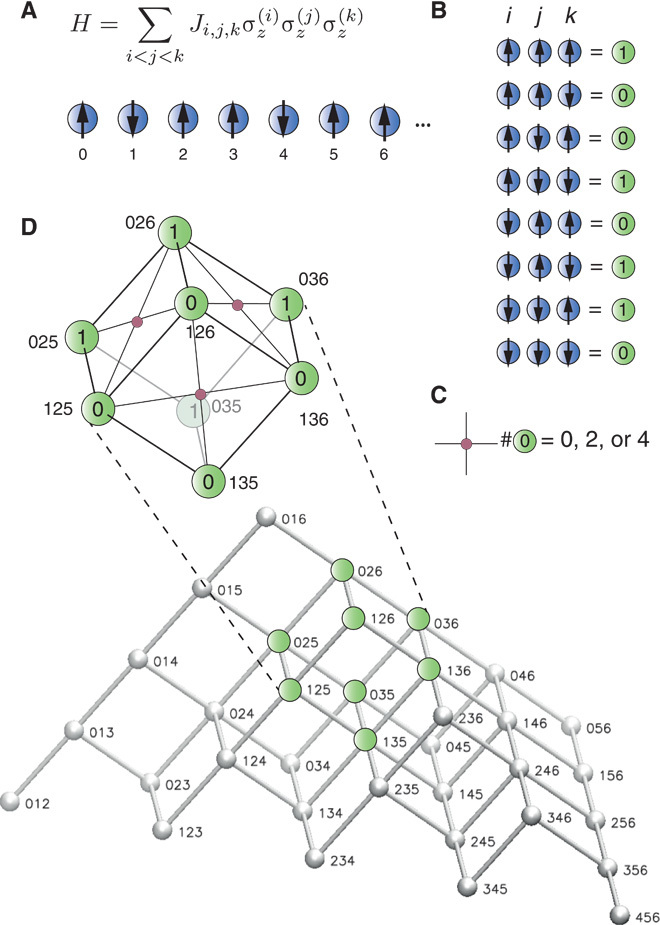
Higher-order interactions. (**A**) Generalization to three-body interaction terms. (**B** and **C**) The translation table (B) from three-body configurations to two-level systems, together with constraints (C), allows for a mapping to a three-dimensional cubic lattice. (**D**) The physical qubits are aligned in a pyramid-slice configuration. Each side of each cube consists of triples, where one index is identical for all four corners. The constraints, identical to that of the pair interaction case, act on the four spins of each side in a face-centered cubic geometry.

The construction of the programmable model for these interactions is depicted in Fig. 4. In analogy to Fig. 1, physical qubits represent the results of the *K*_3_ = *N*(*N* − 1)(*N* − 2)∕6 three-body interactions and are mapped back to the logical degrees of freedom by constraints. The translation table is given in Fig. 4B. The physical qubits can be arranged in a three-dimensional cubic lattice, depicted in Fig. 4D, with the same constraints as given in Eq. 4 but acting now on the four spins of each face of each cube of the lattice. Note that the spins in the triangles have to be taken care of separately because all constraints should contain an even number of spins. Also, note that the translation table in Fig. 4B is a mapping of three bits to a single bit and, here, the number of constraints is at least *K*_3_ − *N*. In principle, the scheme can be extended to four-body and higher-interaction *k*-body terms. The overhead grows then with *N*^*k*^, but the geometrical arrangement of the constraints becomes less practical.

## CONCLUSIONS

In summary, we have presented a fully connected and fully programmable, scalable quantum annealing architecture. The interactions of the logical qubits are mediated by gauge field constraints that can be realized with local interactions between physical qubits. The optimization problem is encoded in local fields acting on the qubits. This allows one to implement an adiabatic sweep where only local fields need to be tuned. The solution of the problem is encoded in the topology of the physical qubits. The resulting adiabatic sweep is fundamentally different from the sweep based on a spin glass (Eq. 1) and allows the introduction of various additional experimental knobs. The dynamics of the encoded logical qubits during the sweep compared to a fictitious all-to-all connected spin glass is an important open question. The architecture may also open opportunities to gain new insights into open challenges in quantum annealing such as the scaling of the minimal gap, an intrinsic error correction, the scaling of quantum fluctuations during the sweep, the role of finite temperatures, and errors from imperfections in the device. The presented architecture, with qubits on a square-lattice geometry in combination with nearest-neighbor interactions, may serve as a blueprint for quantum annealers in various frameworks ranging from superconducting qubits to ultracold gases in optical lattices.

## SUPPLEMENTARY MATERIALS

Supplementary material for this article is available at http://advances.sciencemag.org/cgi/content/full/1/9/e1500838/DC1 Static error from finite constraint strength Fig. S1. Static error. Energy spectrum during the adiabatic optimization Fig. S2. Success probability. Dynamics Special cases Fig. S3. Special cases.
